# Prevalence and factors related to hypouricemia and hyperuricemia in schoolchildren: results of a large-scale cross-sectional population-based study conducted in Japan

**DOI:** 10.1038/s41598-022-19724-1

**Published:** 2022-10-25

**Authors:** Yuhei Aoki, Tadashi Sofue, Ryo Kawakami, Takashi Ozaki, Masahiro Manabe, Kanae Kanda, Takeshi Yoda, Takashi Kusaka, Tomohiro Hirao, Tetsuo Minamino

**Affiliations:** 1 Department of Cardiorenal and Cerebrovascular Medicine, Faculty of Medicine, Kagawa University, 1750-1 Ikenobe, Miki-Chou, Kita-Gun, Kagawa Japan; 2 Department of Pediatrics, Mitoyo-Kanonji Medical Association, Mitoyo, Kagawa Japan; 3 Department of Pediatrics, Kids Medical Manabe, Takamatsu, Kagawa Japan; 4 Department of Public Health, Faculty of Medicine, Kagawa University, Kita, Kagawa Japan; 5 Department of Health and Sports Science, Faculty of Health Science and Technology, Kawasaki University of Medical Welfare, Kurashiki, Okayama Japan; 6 Department of Pediatrics, Faculty of Medicine, Kagawa University, Kita, Kagawa Japan

**Keywords:** Kidney diseases, Obesity, Paediatric research, Epidemiology, Disease prevention, Paediatrics, Acute kidney injury, Dyslipidaemias, Metabolic syndrome, Obesity, Pre-diabetes

## Abstract

Hypouricemia in children including renal hypouricemia, which is a major cause of exercise-induced acute renal injury (EIAKI), is an important clinical problem, in addition to hyperuricemia. However, no large-scale studies of serum uric acid (UA) concentrations in the general pre-adolescent population have been carried out. We conducted a population-based cross-sectional study to measure the prevalences of hypouricemia and hyperuricemia and identify the associated factors. We analyzed 31,822 (16,205 boys and 15,617 girls) 9–10-year-old children who underwent pediatric health check-ups in Kagawa prefecture between 2014 and 2018. Hypouricemia and hyperuricemia were defined using serum UA concentrations of ≤ 2.0 mg/dL and ≥ 6.0 mg/dL, respectively. The prevalence of hypouricemia was 0.38% in both 9- and 10-year-old boys and girls, and was not significantly associated with age, sex, or environmental factors, including overweight. The prevalence of hyperuricemia was significantly higher in boys (2.7%) than in girls (1.9%), and was significantly associated with age, overweight, future diabetes risk, hypertriglyceridemia, low high-density lipoprotein-cholesterol, and liver damage, but not with high low-density lipoprotein cholesterol. Therefore, some pre-adolescent children in the general population in Japan showed hypouricemia. A means of identifying children with hypouricemia and lifestyle guidance measures for the prevention of EIAKI should be established.

## Introduction

Abnormal serum uric acid (UA) concentration in children is considered a biochemical disorder with no clinical significance. However, recent evidence has suggested that both hypouricemia and hyperuricemia in children are important clinical problems^1–5^. Several clinical studies have shown that renal hypouricemia is a risk factor for exercise-induced acute renal injury (EIAKI)^1–3,6–8^, urolithiasis^1,6,7^, and kidney dysfunction^2,3^. Meanwhile, other studies have shown that hyperuricemia in children is related to metabolic syndrome in children^4^ and a predictor of future hypertension^5^.

EIAKI, which represents a major cause of kidney dysfunction associated with hypouricemia, mainly in Asia, develops after repetitive anaerobic exercise (e.g., 100-m runs) and is often associated with pre-exercise cold symptoms and the use of oral antipyretics/analgesics^1^. EIAKI onset was reported to occur between 10 and 54 years of age, with a mean age (standard deviation [SD]) of 21.7 (7.8) years^1^. Therefore, diagnosis of hypouricemia before the age of onset for EIAKI and provision of lifestyle guidance are likely to reduce the risk of incident EIAKI. Because the serum UA concentration in children changes with growth, it is important to establish age- and sex-specific reference values for children. However, hypouricemia in children is conventionally defined as serum UA ≤ 2.0 mg/dL throughout childhood, as in adults^1–3,9,10^. Although hyperuricemia is defined as serum UA ≥ 7.1 mg/dL in adults, there is no universally accepted threshold for the definition of hyperuricemia in children and adolescents. Therefore, previous studies have used a range of cut-off values to define hyperuricemia in children and adolescents^4,11,12^.

To date, no reference values have been established for serum UA concentrations in childhood because there have been no large-scale studies of serum UA concentrations in the general pediatric population. Here, we conducted a population-based epidemiological study as a preparatory step before a precise classification study including genetic analysis. In the study, we aimed to 1) determine the distribution of serum UA concentrations and the prevalences of hypouricemia and hyperuricemia in healthy Japanese schoolchildren aged 9 and 10 years, and 2) identify the factors associated with hypouricemia and hyperuricemia, using data obtained from large-scale population-based pediatric health examinations conducted in Japan.

## Methods

### Study design and population

In this population-based, cross-sectional study, we retrospectively analyzed data for 31,822 participants who had a complete set of data available from Kagawa pediatric health check-ups between 2014 and 2018 in ten municipalities within Kagawa prefecture (Takamatsu, Marugame, Mitoyo, Kanonji, Sakaide, Zentsuji, Tonosho, Miki, Ayagawa, and Kotohira). There were 41,927 fourth-grade children in Kagawa prefecture between 2014 and 2018, meaning that we surveyed 76% of children aged 9–10 years. Kagawa pediatric health check-ups have been conducted in all fourth-grade children living in Kagawa prefecture, Japan, since 2012^13^. Given that there were 36,410 fourth-grade children in the ten municipalities among the total of 41,927 fourth-grade children in Kagawa Prefecture between 2014 and 2018, the fourth-grade children in these ten municipalities covered 87% of all children at that age in the prefecture. Of the 36,410 children in the ten municipalities, 34,169 (94%) children underwent pediatric check-ups between 2014 and 2018.

In the Kagawa pediatric health check-ups, fasting blood samples were collected, the height and body mass of the children were measured by trained staff, and a self-administered questionnaire was completed regarding their lifestyle. Serum UA concentration was measured using an enzymatic method. The distribution of serum UA concentrations was evaluated using histograms for each sex and age, and the prevalences of hypouricemia and hyperuricemia were evaluated for each sex and age. Hypouricemia was defined as serum UA ≤ 2.0 mg/dL, based on the Japanese clinical practice guideline for renal hypouricemia^10^ and previous studies^1–3,9^, because there is no practical consensus regarding the absolute serum UA concentration that represents hypouricemia in school-age children^4,11,12^. To evaluate the characteristics of the participants with hypouricemia, we assigned the 31,822 participants to two subgroups: those with hypouricemia (n = 122) and those without (n = 31,700). To assess the accuracy of the criteria for hypouricemia, the characteristics of the participants with UA ≤ 2.5 mg/dL, which represents the 2.5 percentile of the cohort, and the participants with serum UA ≤ 1.0 mg/dL were evaluated and compared with those of the participants with hypouricemia. Given that the cohort comprised school-age children of a specific age, we defined hyperuricemia as serum UA ≥ 6.0 mg/dL, which represents the 97.5th percentile of the cohort. To evaluate the characteristics of the participants with hyperuricemia, we assigned the 31,822 participants to two further subgroups: those with hyperuricemia (n = 734) and those without (n = 31,088). To assess the accuracy of the criteria for hyperuricemia, the characteristics of the participants with UA ≥ 7.0 mg/dL were evaluated and compared with those of the participants with hyperuricemia.

Overweight and underweight was evaluated using the obesity score for Japanese schoolchildren^14^. Overweight and underweight were defined as obesity scores of ≥ 20% and ≤ –20%, respectively. Hemoglobin A1c (HbA1c) levels were presented as National Glycohemoglobin Standardization Program equivalent values. In adults, HbA1c ≥ 5.6% was reported to indicate a significant risk of developing diabetes^15^, but there are no established criteria for assessment of future diabetes risk in school-age children; therefore, we used HbA1c ≥ 5.6% as an indicator of future diabetes risk. Hypertriglyceridemia, hyper-low-density lipoprotein (LDL)-cholesterolemia, and hypo-high-density lipoprotein (HDL)-cholesterolemia were defined as triglyceride ≥ 140 mg/dL, LDL-cholesterol ≥ 140 mg/dL, and HDL-cholesterol ≤ 40 mg/dL, respectively, according to the Japan Atherosclerosis Society guidelines^16^. Liver damage was defined as activity of one among aspartate aminotransferase (AST), alanine aminotransferase (ALT), or γ-GTP (glutamyl transpeptidase) above the 97.5th percentile of the corresponding reference range for 9- and 10-year-old Asian children^17^.

### Ethics statement

The study was conducted in accordance with the principles of the World Medical Association Declaration of Helsinki. The study was approved by the Ethics Committee of Kagawa University (#2020–020) and all relevant local governments and medical associations. Because all the data were fully anonymized before being accessed, informed consent was waived in favor of the possibility of opting out by contacting the city hall of each municipality. A waiver of the need for informed consent was approved by the Kagawa University Ethics Committee. All the data were stored in a protected computer database. One independent author had full access to all the data and take complete responsibility for the integrity of the data and the accuracy of the data analysis.

### Statistical analysis

Continuous variables are shown as medians with interquartile intervals, means with SDs, or counts with percentages, as appropriate. The distributions of variables were evaluated using histograms, quantile–quantile plots, and the Kolmogorov–Smirnov test. Clinical data were compared between groups using the chi-square test for categorical variables, and Student’s *t*-test, one-way analysis of variance, two-way analysis of variance, or the Mann–Whitney *U*-test for continuous variables. The data were analyzed using JMP® PRO 15 (SAS Institute Inc., Cary, NC, USA), and values of *p* < 0.05 were considered to indicate statistical significance.

To evaluate the effect of individual factors on hypouricemia, univariate analyses were performed using the Pearson chi-square test for age and sex, and Fisher’s exact test for other variables. To determine the effect of individual factors on hyperuricemia, univariate analyses were conducted for age, sex, obesity score, future diabetes risk, hypertriglyceridemia, hyper-LDL-cholesterolemia, hypo-HDL-cholesterolemia, and liver damage using the Pearson chi-square test. To investigate the effect of individual factors on serum UA ≤ 2.5 mg/dL, univariate analyses were carried out using Fisher’s exact test for hypo-HDL-cholesterolemia, and the Pearson chi-square test for other variables. To clarify the effect of individual factors on serum UA ≥ 7.0 mg/dL, univariate analyses were performed using the Pearson chi-square test for age, sex, obesity score, future diabetes risk, hypertriglyceridemia, hyper-LDL-cholesterolemia, hypo-HDL-cholesterolemia, and liver damage.

To identify factors associated with hypouricemia, hyperuricemia, serum UA ≤ 2.5 mg/dL, and serum UA ≥ 7.0 mg/dL, multiple logistic regression analysis was conducted, using age, sex, and obesity score, or future diabetes risk, hypertriglyceridemia, hyper-LDL-cholesterolemia, hypo-HDL-cholesterolemia, and liver damage as covariates.

## Results

### Characteristics of the enrolled participants

The characteristics of the participants are shown in Table 1. Of the 31,822 participants, 51% were male and 49% were female. Furthermore, 52% were 9 years of age and 48% were 10 years of age. The mean height (SD) was 135.1 (6.2) cm and the mean body mass (SD) was 31.3 (6.5) kg. Among the total participants, 9.5% were overweight and 2.5% were underweight. Future diabetes risk, high circulating LDL-cholesterol concentration, and liver damage were identified in 9.6%, 3.6%, and 12.0% of the participants, respectively.

**Table 1:** Characteristics of the study participants, categorized according to sex.

	Full cohort	Boys	Girls
n	31,822	16,205	15,617
**Age, n (%)**			
9 years	16,444 (52)	8,347 (52)	8,097 (52)
10 years	15,378 (48)	7858 (48)	7520 (48)
Height (cm)	135.1 (6.2)	134.9 (5.8)	135.4 (6.5)
Body mass (kg)	31.3 (6.5)	31.6 (6.7)	31.1 (6.3)
Body mass index (kg/m^2^)	17.0 (2.6)	17.2 (2.8)	16.8 (2.4)
**Obesity score, n (%)**			
Overweight	3,018 (9.5)	1,747 (10.8)	1,271 (8.1)
Normal	28,009 (88)	14,067 (86.8)	13,942 (89.3)
Underweight	795 (2.5)	391 (2.4)	404 (2.6)
HbA1c (%)	5.3 (0.2)	5.3 (0.2)	5.3 (0.2)
Future diabetes risk, n (%)	3062 (9.6)	1650 (10.2)	1412 (9.0)
Triglyceride (mg/dL)	68 (40)	66 (38)	70 (42)
Hypertriglyceridemia, n (%)	1404 (4.4)	717 (4.4)	687 (4.4)
LDL-cholesterol (mg/dL)	95 (23)	94 (23)	97 (23)
Hyper-LDL-cholesterolemia, n (%)	1154 (3.6)	515 (3.2)	639 (4.1)
HDL-cholesterol (mg/dL)	65 (13)	66 (13)	64 (13)
Hypo-HDL-cholesterolemia, n (%)	334 (1.0)	166 (1.0)	168 (1.1)
AST (IU/L)	26 (6)	27 (7)	25 (6)
ALT (IU/L)	15 (11)	16 (14)	13 (8)
γ-GTP (IU/L)	14 (6)	15 (7)	13 (3)
Liver damage, n (%)	3808 (12.0)	2189 (13.5)	1619 (10.4)

Data are shown as mean (standard deviation) unless otherwise indicated.

LDL, low-density lipoprotein; HDL, high-density lipoprotein; AST, aspartate transaminase; ALT, alanine transaminase; γ-GTP, gamma glutamyl transpeptidase.

### Distributions of serum UA concentrations and prevalences of hypouricemia and hyperuricemia

The mean, median and central 95th and 99th percentile interval of the serum UA concentrations are shown in Table 2. The mean (SD) serum UA concentration in all participants was 4.11 (0.84) mg/dL, and that in boys was significantly higher than that in girls (*p* < 0.01). Among 10-year-old children, the mean serum UA concentration in boys was significantly higher than that in girls. The mean serum UA concentration in 10-year-old participants was significantly higher than that in 9-year-old participants (*p* < 0.01). The median [interquartile range], central 95th percentile interval, and central 99th percentile interval of the full cohort were 4.1 [3.6, 4.6] mg/dL, 2.6–5.9 mg/dL, and 2.1–6.7 mg/dL, respectively.

**Table 2:** The mean, median and central 95th and 99th percentile interval of serum uric acid concentrations, categorized according to age and sex.

		n	Mean (SD)	Median [IQR]	Central 95th percentile interval	Central 99th percentile interval
Full cohort	Full cohort	31,822	4.11 (0.84)	4.1 [3.6, 4.6]	2.6–5.9	2.1–6.7
	Boys	16,205	4.13 (0.86)	4.1 [3.6, 4.6]	2.5–6.0	2.1–6.8
	Girls	15,617	4.09 (0.81)	4.0 [3.6, 4.6]	2.6–5.8	2.1–6.5
9-year-olds	Full cohort	16,444	4.08 (0.82)	4.0 [3.5, 4.6]	2.6–5.8	2.1–6.5
	Boys	8,347	4.09 (0.83)	4.0 [3.5, 4.6]	2.6–5.9	2.1–6.7
	Girls	8,097	4.07 (0.80)	4.0 [3.5, 4.5]	2.6–5.8	2.1–6.4
10-year-olds	Full cohort	15,378	4.14 (0.85)†	4.1 [3.6, 4.7]	2.6–6.0	2.1–6.7
	Boys	7,858	4.17 (0.88)	4.1 [3.6, 4.7]	2.5–6.1	2.1–6.8
	Girls	7,520	4.11 (0.82)*	4.1 [3.6, 4.6]	2.6–5.9	2.1–6.6

*p < 0.05 *vs*. boys; †*p* < 0.05 *vs*. 9-year-olds.

SD, standard deviation; IQR, interquartile range.

**Figure 1:**
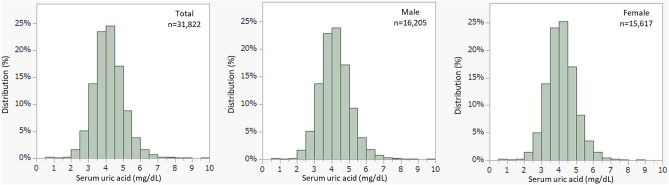
Distribution of serum uric acid concentrations in children aged 9–10 years. (**a**) Full cohort, (**b**) male participants, and (**c**) female participants. Hypouricemia and hyperuricemia were identified in 0.38% and 2.3% of the full cohort, respectively.

The distributions of the serum UA concentrations are shown in Fig. 1 and Table 3. Among the 31,822 participants, hypouricemia (serum UA ≤ 2 mg/dL) was identified in 122 (0.38%) participants (57 boys and 65 girls). Hyperuricemia (serum UA ≥ 6.0 mg/dL) was identified in 734 (2.3%) participants (437 boys and 297 girls). No significant difference in the prevalence of hypouricemia was found between boys (0.35%) and girls (0.42%) (*p* = 0.35). The prevalence of hyperuricemia was significantly higher in boys (2.7%) than girls (1.9%) (*p* < 0.01).

**Table 3:** Distribution of serum uric acid concentrations.

		Boys	Girls	9-year-olds	10-year-olds	Full cohort
≤ 2.0 mg/dL	n (%)	57 (0.35)	65 (0.41)	65 (0.40)	57 (0.37)	122 (0.38)
2.1–3.0 mg/dL	n (%)	1,356 (8.4)	1,257 (8.0)	1,380 (8.4)	1,233 (8.0)	2,613 (8.2)
3.1–4.0 mg/dL	n (%)	6,405 (39.5)	6,494 (41.6)	6,883 (41.9)	6,016 (39.0)	12,899 (40.5)
4.1–5.0 mg/dL	n (%)	6,245 (38.5)	6,035 (38.6)	6,280 (38.2)	6,000 (39.0)	12,280 (38.6)
5.1–5.9 mg/dL	n (%)	1,705 (10.5)	1,469 (9.4)	1,499 (9.1)	1,675 (10.9)	3,174 (10.0)
≥ 6.0 mg/dL	n (%)	437 (2.7)	297 (1.9)	337 (2.0)	397 (2.6)	734 (2.3)

### Characteristics of the participants with hyperuricemia or hypouricemia

The characteristics of the participants with hypouricemia are shown in Table 4. The prevalence of hypouricemia in the full cohort was 0.38% (122 of 31,822). The age distribution and sex ratio were similar in the participants with and without hypouricemia. Participants with hypouricemia had similar prevalences of overweight, future diabetes risk, hypertriglyceridemia, hyper-LDL-cholesterolemia, and low circulating HDL-cholesterol, but had lower prevalence of liver damage (*p* < 0.01) than those without. Serum UA ≤ 2.5 mg/dL was identified in 764 (2.4%) participants (413 boys [2.5%] and 351 girls [2.2%]) (Supplementary Table S1). Serum UA ≤ 1.0 mg/dL was identified in 42 (0.132%) participants (22 boys [0.136%] and 20 girls [0.128%]).

**Table 4:** Characteristics of the participants with hypouricemia.

	Hypouricemia	No hypouricemia	*p*-value
n	122	31,700	
Age of 10 years, n (%)	57 (47)	15,321 (48)	0.72
Female sex, n (%)	65 (53)	15,552 (49)	0.35
**Obesity score, n (%)**			0.22
Overweight	6 (4.9)	3,012 (9.5)	
Normal	113 (93)	27,896 (88)	
Underweight	3 (2.5)	792 (2.5)	
Future diabetes risk, n (%)	7 (5.7)	3,055 (9.6)	0.17
Hypertriglyceridemia, n (%)	4 (3.3)	1,400 (4.4)	0.82
Hyper-LDL-cholesterolemia, n (%)	2 (1.6)	1,152 (3.6)	0.33
Hypo-HDL-cholesterolemia, n (%)	0 (0)	334 (1.1)	0.64
Liver damage, n (%)	5 (4.1)	3,803 (12)	< 0.01*

*p < 0.05 *vs*. no hypouricemia.

LDL, low-density lipoprotein; HDL, high-density lipoprotein.

The characteristics of the participants with hyperuricemia are shown in Table 5. Participants with hyperuricemia had significantly higher percentages of being 10 years of age (54%, *p* < 0.01) and boys (60%, *p* < 0.01) than those without. The prevalences of overweight (47%, *p* < 0.01), future diabetes risk (18%, *p* < 0.01), hypertriglyceridemia (18%, *p* < 0.01), hyper-LDL-cholesterolemia (8.9%, *p* < 0.01), hypo-HDL-cholesterolemia (4.5%, *p* < 0.01), and liver damage (42%, *p* < 0.01) were significantly higher in participants with hyperuricemia than in those without. Serum UA ≥ 7.0 mg/dL was identified in 78 (0.25%) participants (54 boys [0.33%] and 24 girls [0.15%]) (Supplementary Table S2).

**Table 5:** Characteristics of the participants with hyperuricemia.

	Hyperuricemia	No hyperuricemia	*p*-value
n	734	31,088	
Age of 10 years, n (%)	397 (54)	14,981 (48)	< 0.01*
Female sex, n (%)	297 (40)	15,320 (49)	< 0.01*
**Obesity score, n (%)**			< 0.01*
Overweight	344 (47)	2,674 (8.6)	
Normal	388 (53)	27,621 (89)	
Underweight	2 (0.3)	793 (2.6)	
Future diabetes risk, n (%)	130 (18)	2,932 (9.4)	< 0.01*
Hypertriglyceridemia, n (%)	106 (14)	1,298 (4.2)	< 0.01*
Hyper-LDL-cholesterolemia, n (%)	65 (8.9)	1,089 (3.5)	< 0.01*
Hypo-HDL-cholesterolemia, n (%)	33 (4.5)	301 (1.0)	< 0.01*
Liver damage, n (%)	308 (42)	3,500 (11)	< 0.01*

*p < 0.05 *vs*. no hyperuricemia.

LDL, low-density lipoprotein; HDL, high-density lipoprotein.

### Factors associated with hypouricemia or hyperuricemia

The factors associated with hypouricemia are shown in Table 6. According to the results of the multiple logistic regression analysis, age, sex, obesity score, future diabetes risk, hypertriglyceridemia, hyper-LDL-cholesterolemia, and hypo-HDL-cholesterolemia were not significantly associated with the prevalence of hypouricemia, while liver damage was negatively associated with the prevalence of hypouricemia (adjusted odds ratio [AOR] 0.36 [95% confidence interval [CI] 0.15–0.90]). Age, future diabetes risk, hypertriglyceridemia, hyper-LDL-cholesterolemia, and hypo-HDL-cholesterolemia were not significantly associated with the prevalence of serum UA ≤ 2.5 mg/dL, while female sex (AOR 0.86 [95% CI 0.74–0.99]), overweight (AOR 0.30 [95% CI 0.19–0.46]), and liver damage (AOR 0.71 [95% CI 0.54–0.94]) were negatively associated with the prevalence of serum UA ≤ 2.5 mg/dL (Supplementary Table S3).

**Table 6:** Factors associated with hypouricemia.

	Univariate analysis	Multivariate analysis
	Odds ratio [95% CI]	Adjusted odds ratio [95% CI]
Age of 10 years	0.94 [0.66–1.34]	0.94 [0.66–1.34]
Female sex	1.18 [0.83–1.69]	1.15 [0.81–1.65]
Obesity score: Normal	1	1
Overweight	0.49 [0.22–1.12]	0.67 [0.29–1.54]
Underweight	0.94 [0.30–2.95]	0.92 [0.29–2.91]
Future diabetes risk	0.57 [0.27–1.23]	0.61 [0.29–1.32]
Hypertriglyceridemia	0.73 [0.27–1.99]	0.93 [0.34–2.54]
Hyper-LDL-cholesterolemia	0.44 [0.11–1.79]	0.52 [0.13–2.10]
Liver damage	0.31 [0.13–0.77]	0.36 [0.15–0.90]

CI, confidence interval; LDL, low-density lipoprotein.

Adjusted odds ratios and 95% CIs were calculated by logistic regression analysis using age, sex, and obesity score, future diabetes risk, hypertriglyceridemia, hyper-LDL-cholesterolemia, and liver damage as covariates.

The factors associated with hyperuricemia are shown in Table 7. In the multiple logistic regression analysis, the prevalence of hyperuricemia was significantly associated with age of 10 years (AOR 1.27 [95% CI 1.09–1.47]), female sex (AOR 0.83 [95% CI 0.71–0.96]), overweight (AOR 5.49 [95% CI 4.62–6.52]), underweight (AOR 0.18 [95% CI 0.05–0.74]), future diabetes risk (AOR 1.43 [95% CI 1.16–1.75]), hypertriglyceridemia (AOR 1.47 [95% CI 1.16–1.88]), hypo-HDL-cholesterolemia (AOR 1.82 [95% CI 1.21–2.75]), and liver damage (AOR 2.72 [95% CI 2.28–3.23]), but was not significantly associated with hyper-LDL-cholesterolemia. Age, future diabetes risk, hypertriglyceridemia, and hyper-LDL-cholesterolemia were not significantly associated with the prevalence of serum UA ≥ 7.0 mg/dL, while overweight (AOR 9.93 [95% CI 5.74–17.17]), HDL-cholesterolemia (AOR 3.93 [95% CI 1.80–8.60]), and liver damage (AOR 3.20 [95% CI 1.91–5.37]) were associated with the prevalence of serum UA ≥ 7.0 mg/dL, and female sex (AOR 0.86 [95% CI 0.74–0.99]) was negatively associated with the prevalence of serum UA ≥ 7.0 mg/dL (Supplementary Table S4).

**Table 7:** Factors associated with hyperuricemia.

	Univariate analysis	Multivariate analysis
	Odds ratio [95% CI]	Adjusted odds ratio [95% CI]
Age of 10 years	1.27 [1.09–1.47]	1.27 [1.09–1.47]
Female sex	0.70 [0.60–0.81]	0.83 [0.71–0.96]
Obesity score: Normal	1	1
Overweight	9.16 [7.88–10.65]	5.49 [4.62–6.52]
Underweight	0.18 [0.04–0.72]	0.18 [0.05–0.74]
Future diabetes risk	2.07 [1.70–2.51]	1.43 [1.16–1.75]
Hypertriglyceridemia	3.87 [3.13–4.79]	1.47 [1.16–1.88]
Hyper-LDL-cholesterolemia	2.68 [2.06–3.48]	1.26 [0.95–1.67]
Hypo-HDL-cholesterolemia	4.82 [3.34–6.95]	1.82 [1.21–2.75]
Liver damage	5.70 [4.90–6.63]	2.72 [2.28–3.23]

CI, confidence interval; LDL, low-density lipoprotein; HDL, high-density lipoprotein.

Adjusted odds ratios and 95% CIs were calculated by logistic regression analysis using age, sex, and obesity score, future diabetes risk, hypertriglyceridemia, hyper-LDL-cholesterolemia, hypo-HDL-cholesterolemia, and liver damage as covariates.

## Discussion

In this study, we have demonstrated for the first time the prevalences of hypouricemia and hyperuricemia, and the factors associated with each, in school-age children in Japan using a population-based cross-sectional study. The most important finding of the study is that the prevalence of hypouricemia in school-age children is as high as 0.38%. The prevalence of hypouricemia in our study cohort is consistent with previously reported prevalences in Japanese adults (0.2% in men and 0.4% in women)^2^ and Japanese children aged 9–15 years (0.22% in boys and 0.25% in girls)^9^. The prevalence of hypouricemia was reported to show regional differences in Japan^3^, which may be due to differences in the prevalence of genetic variants. Mass screening of the adult general population may help to clarify differences between the prevalences of hypouricemia in adults and school-age children.

Hypouricemia can be caused by hyperexcretion of UA, renal hypouricemia, and Fanconi syndrome, or by lower uric acid production, including that associated with xanthinuria, molybdenum cofactor deficiency, purine nucleoside phosphorylase deficiency, phosphoribosyl pyrophosphate synthetase hypoactivity, severe hepatic injury, and emaciation^10^. In the present study, we defined hypouricemia in school-age children as serum UA ≤ 2.0 mg/dL, which is the cut-off value for adults^10^. Our results showed no significant associations between the prevalence of hypouricemia and environmental factors, including body size and lifestyle diseases. On the basis of these findings, which are similar to those in adults, the prevalence of hypouricemia in school-age children appears to be mainly influenced by genetic factors (i.e., hypouricemia due to renal hypouricemia [RHUC]), rather than environmental factors. Therefore, we consider that use of the same definition for hypouricemia in school-age children to that used in adults is appropriate. Furthermore, when hypouricemia was defined as serum UA ≤ 2.5 mg/dL, which represents the 2.5th percentile of the cohort, the prevalence of hypouricemia was associated with female sex and environmental factors (such as overweight). Therefore, we considered it better to define serum UA ≤ 2.0 mg/dL based on the clinical practice guideline to screen for renal hypouricemia, which is influenced by genetic factors.

The causes of RHUC type 1 and type 2 were identified as genetic mutations in *URAT1*/*SLC22A12* and *GLUT9*/*SLC2A9*, respectively^18,19^. The majority of RHUC cases are type 1^6^. The urinary UA excretion rates in RHUC type 1 and type 2 were reported to be 30–70% and > 100%, respectively, whereas the normal range is 5.5–11.1%^20^. In some adult patients with RHUC type 1, a heterozygous mutation is associated with mild hypouricemia (serum UA ≥ 2.1 mg/dL)^21^. However, EIAKI has even been reported in patients with mild hypouricemia caused by such heterozygous mutations^22,23^. Because serum UA concentrations can be high in adults because of overweight, alcohol consumption, or kidney dysfunction, the serum UA concentration may be ≥ 2.1 mg/dL, even in patients with RHUC type 1 heterozygous mutations. Therefore, mass screening of school-age children may provide more accurate risk stratification because it involves the assessment of fewer environmental factors.

An association between hypouricemia and kidney dysfunction, which may be mediated by EIAKI, was previously reported^2^. Hypouricemia is a major risk factor for EIAKI: 21.1% of patients with RHUC have a history of EIAKI^7^ and 57.1% of patients with EIAKI have RHUC^1^. In a systematic review, the mean age at onset of EIAKI was calculated to be 21.7 years^1^. Because hypouricemia is asymptomatic, most people develop EIAKI without knowing that they originally had risk factors. The school-age children investigated here were younger than the vast majority of people with EIAKI, and therefore the risk of subsequent EIAKI may be reduced by providing guidance to this age group. Thus, the present findings may permit stratification of the risk of EIAKI using the presence of hypouricemia.

It has been reported that the prevalence of hypouricemia decreases with age in women^2^; this is because estrogen functions to reduce the serum UA concentration and the effect disappears with the onset of menopause. In the present study, there was no sex difference in the prevalence of hypouricemia or the prevalence of serum UA ≤ 1.0 mg/dL in school-age children, possibly because of the low circulating estrogen concentrations in girls at 9–10 years of age, but the mean serum UA concentration was higher in boys than girls at 10 years of age. In contrast, a previous study showed a sex difference in serum UA in Japanese children at 11 years of age^9^, which may have arisen, at least in part, because overweight and liver damage are more prevalent in boys than in girls and because of the influence of estrogen.

We also found that the prevalence of hyperuricemia in school-age boys was higher than that in girls. Lifestyle factors, being 10 years of age, and being male were associated with the prevalence of hyperuricemia, while hyper-LDL-cholesterolemia was not. An association between hyperuricemia and metabolic syndrome was found in pre-adolescents in small-scale studies conducted in Japan^12^ and China^24^, as well as in adults^25^ and adolescents^4^. However, this is the first report on the prevalence of hyperuricemia and its associated factors in school-age children. In the present study, hyperuricemia (serum UA ≥ 6.0 mg/dL) and serum UA ≥ 7.0 mg/dL were significantly associated with factors related to metabolic syndrome, such as overweight and high glycated hemoglobin level, as well as with liver damage, which was likely secondary to non-alcoholic fatty liver disease. These findings suggest that, in contrast to hypouricemia, pediatric hyperuricemia is more greatly influenced by environmental factors than by genetic factors. To detect genetic hyperuricemia accurately, a combination of health checkups and genetic examination may be useful, and we are in the process of establishing a system with genetic examination.

In the present study, 3.6% of the children had hyper-LDL-cholesterolemia. A previous cross-sectional study of adults found a positive correlation between hyperuricemia and hyper-LDL-cholesterolemia^26^, but such a correlation has not been reported in children. The present results suggest that hyper-LDL-cholesterolemia in children mainly results from genetic factors (i.e., familial hypercholesterolemia [FH] and familial combined hyperlipidemia [FCHL]). A previous randomized controlled trial showed that statin treatment of pediatric FH patients slowed the progression of atherosclerosis^27^. Therefore, pediatric mass screening represents an excellent strategy for early diagnosis of FH/FCHL and prevention of coronary artery disease.

The present study had several limitations. First, because it was a cross-sectional study, it was not possible to show a cause-and-effect relationship. Second, an accurate diagnosis of the cause of hypouricemia in children could not be made because of the lack of genetic testing and knowledge regarding the use of oral medications or presence of systemic diseases. Third, the sample was not highly representative of the wider population. We surveyed 76% of children aged 9–10 years in Kagawa Prefecture; therefore, the sample should be representative of the population of this prefecture. However, it is unclear whether our results can be applied to Japan as a whole or to other countries. Meanwhile, the study had a number of strengths. First, to the best of our knowledge, this was the first study to determine the prevalence of hypouricemia in school-age Japanese children. Second, there was unlikely to be selection bias, random errors, or many confounding factors because we surveyed a large sample of the general population, although we could not control for all variables that may affect UA concentrations, such as diets rich in red meat and sugar-sweetened beverages. Third, because we investigated children with a narrow age range, we were able to analyze the associations with serum UA concentrations without a confounding effect of age.

In conclusion, we have shown that the prevalence of hypouricemia in school-age children is consistent with the prevalence in adults and is not influenced by environmental factors. Therefore, it is reasonable to define serum UA ≤ 2.0 mg/dL as hypouricemia in children as well as in adults. We plan to recommend the medical examination of children with hypouricemia including genetic examination to ensure that an accurate diagnosis is made and to establish appropriate lifestyle guidance for the prevention of EIAKI. Further longitudinal studies are needed to determine the long-term prognosis of patients with hypouricemia and the effectiveness of interventions.

## Supplementary Information

Supplementary Information.

## Data Availability

The data that support the findings of this study are available from Takamatsu City, Marugame City, Mitoyo City, Kanonji City, Sakaide City, Zentuji City, Tonosho Cho, Miki Cho, Ayagawa Cho, and Kotohira Cho; however, restrictions apply to the availability of these data, which were used under license for the present study and are not publicly available. Nevertheless, data are available from the authors upon reasonable request and with permission from Takamatsu City, Marugame City, Mitoyo City, Kanonji City, Sakaide City, Zentuji City, Tonosho Cho, Miki Cho, Ayagawa Cho, and Kotohira Cho.
