# Supplementary material for: Prevalence and factors related to hypouricemia and hyperuricemia in schoolchildren: results of a large-scale cross-sectional population-based study conducted in Japan
Source: Sci Rep. 2022 Oct 25;12:17848. doi: 10.1038/s41598-022-19724-1 (PMC9596694; doi:10.1038/s41598-022-19724-1)
Supplement: Supplementary file 1 — Supplementary Information. [file 41598_2022_19724_MOESM1_ESM.docx]

**Prevalence and factors related to hypouricemia and hyperuricemia in schoolchildren: Results of a** **large-scale cross-sectional population-based study conducted in Japan**

Yuhei Aoki^1^, Tadashi Sofue^1^, Ryo Kawakami^1^, Takashi Ozaki^2^, Masahiro Manabe^3^, Kanae Kanda^4^, Takeshi Yoda^5^, Takashi Kusaka^6^, Tomohiro Hirao^4^, Tetsuo Minamino*^1^

^1^Department of Cardiorenal and Cerebrovascular Medicine, Faculty of Medicine, Kagawa University, Kagawa, Japan

^2^Department of Pediatrics, Mitoyo&Kanonji City Medical Association, Kagawa, Japan

^3^Department of Pediatrics, Kids Medical Manabe, Kagawa, Japan

^4^Department of Public Health, Faculty of Medicine, Kagawa University, Kagawa, Japan

^5^Department of Health and Sports Science, Faculty of Health Science and Technology, Kawasaki University of Medical Welfare, Okayama, Japan

^6^Department of Pediatrics, Faculty of Medicine, Kagawa University, Kagawa, Japan

**Short Title:** Prevalence of hypouricemia in schoolchildren in Japan

***Corresponding Author**

Tetsuo Minamino, M.D., Ph.D.

Department of Cardiorenal and Cerebrovascular Medicine, Kagawa University, 1750-1 Ikenobe, Miki-Chou, Kida-Gun, Kagawa, Japan.

Tel: +81-87-891-2150;Fax: +81-87-891-2152; E-mail: [minamino.tetsuo.gk@kagawa-u.ac.jp](mailto:minamino.tetsuo.gk@kagawa-u.ac.jp)

Running title: Prevalence of hypouricemia in schoolchildren in Japan.

Key words: mass screening, school-age children, uric acid, hypouricemia, hyperuricemia

Supplementary Table Legends

Supplementary Table S1. Characteristics of the participants with serum UA ≤2.5 mg/dL

Supplementary Table S2. Characteristics of the participants with serum UA ≥7.0 mg/dL

Supplementary Table S3. Factors associated with serum UA ≤2.5 mg/dL

Supplementary Table S4. Factors associated with serum UA ≥7.0 mg/dL

**Supplementary Table S1. Characteristics of the participants with serum UA ≤2.5 mg/dL**

|  | **Participants with UA ≤2.5 mg/dL** | **Participants with UA >2.5 mg/dL** | ***p*-value** |
| --- | --- | --- | --- |
| **n** | 764 | 31,058 |  |
| **Age of 10 years, n (%)** | 372 (49) | 15,006 (48) | 0.84 |
| **Female sex, n (%)** | 351 (46) | 15,266 (49) | 0.08 |
| **Obesity score, n (%)** |  |  | <0.01* |
| Overweight | 21 (2.7) | 2,997 (9.6) |  |
| Normal | 716 (94) | 27,293 (88) |  |
| Underweight | 27 (3.5) | 768 (2.5) |  |
| **Future diabetes risk, n (%)** | 63 (8.2) | 2,999 (9.7) | 0.18 |
| **Hypertriglyceridemia, n (%)** | 29 (3.8) | 1,375 (4.4) | 0.39 |
| **Hyper-LDL-cholesterolemia, n (%)** | 18 (2.4) | 1,136 (3.7) | 0.04* |
| **Hypo-HDL-cholesterolemia, n (%)** | 5 (0.7) | 329 (1.1) | 0.37 |
| **Liver damage, n (%)** | 56 (7.3) | 3,752 (12) | <0.01* |

**p* < 0.05 *vs*. participants with UA >2.5 mg/dL.

Abbreviations: LDL, low-density lipoprotein; HDL, high-density lipoprotein.

**Supplementary Table S2. Characteristics of the participants with serum UA ≤7.0 mg/dL**

|  | **Participants with UA ≥7.0 mg/dL** | **Participants with UA <7.0 mg/dL** | ***p*-value** |
| --- | --- | --- | --- |
| **n** | 78 | 31,744 |  |
| **Age of 10 years, n (%)** | 43 (55) | 15,335 | 0.23 |
| **Female sex, n (%)** | 24 (31) | 16,151 | <0.01* |
| **Obesity score, n (%)** |  |  | <0.01* |
| Overweight | 53 (68) | 2,965 |  |
| Normal | 25 (32) | 27,984 |  |
| Underweight | 0 (0) | 795 |  |
| **Future diabetes risk, n (%)** | 16 (21) | 3,046 | <0.01* |
| **Hypertriglyceridemia, n (%)** | 16 (21) | 1,388 | <0.01* |
| **Hyper-LDL-cholesterolemia, n (%)** | 12 (15) | 1,142 | <0.01* |
| **Hypo-HDL-cholesterolemia, n (%)** | 9 (12) | 325 | <0.01* |
| **Liver damage, n (%)** | 45 (58) | 3,763 | <0.01* |

**p* < 0.05 *vs*. participants with UA <7.0 mg/dL.

Abbreviations: LDL, low-density lipoprotein; HDL, high-density lipoprotein.

**Supplementary Table S3. Factors associated with serum UA ≤2.5 mg/dL**

|  | Univariate analysis | Multivariate analysis |
| --- | --- | --- |
|  | Odds ratio [95% CI] | Adjusted odds ratio [95% CI] |
| **Age of 10 years** | 1.02 [0.88–1.17] | 1.02 [0.88–1.17] |
| **Female sex** | 0.88 [0.76–1.02] | 0.86 [0.74–0.99] |
| **Obesity score:** Normal | 1 | 1 |
| Overweight | 0.27 [0.17–0.41] | 0.30 [0.19–0.46] |
| Underweight | 1.34 [0.91–1.98] | 1.33 [0.90–1.97] |
| **Future diabetes risk** | 0.84 [0.65–1.09] | 0.90 [0.69–1.16] |
| **Hypertriglyceridemia** | 0.85 [0.59–1.23] | 1.12 [0.76–1.63] |
| **Hyper-LDL-cholesterolemia** | 0.64 [0.40–1.02] | 0.77 [0.48–1.23] |
| **Hypo-HDL-cholesterolemia** | 0.62 [0.25–1.49] | 0.78 [0.32–1.91] |
| **Liver damage** | 0.58 [0.44–0.76] | 0.71 [0.54–0.94] |

Abbreviations: CI, confidence interval; LDL, low-density lipoprotein.

Adjusted odds ratios and 95% CIs were calculated by logistic regression analysis using age, sex, and obesity score, future diabetes risk, hypertriglyceridemia, hyper-LDL-cholesterolemia, hypo-HDL-cholesterolemia, and liver damage as covariates.

**Supplementary Table S4. Factors associated with serum UA ≥7.0 mg/dL**

|  | Univariate analysis | Multivariate analysis |
| --- | --- | --- |
|  | Odds ratio [95% CI] | Adjusted odds ratio [95% CI] |
| **Age of 10 years** | 1.31 [0.84–2.06] | 1.27 [0.81–2.00] |
| **Female sex** | 0.46 [0.28–0.74] | 0.59 [0.36–0.97] |
| **Obesity score:** Normal | 1 | 1 |
| Overweight | 20.58 [12.77–33.16] | 9.93 [5.74–17.17] |
| **Future diabetes risk** | 2.43 [1.40–4.22] | 1.33 [0.75–2.35] |
| **Hypertriglyceridemia** | 5.64 [3.25–9.80] | 1.25 [0.67–2.33] |
| **Hyper-LDL-cholesterolemia** | 4.87 [2.63–9.04] | 1.81 [0.95–3.46] |
| **Hypo-HDL-cholesterolemia** | 12.61 [6.24–25.47] | 3.93 [1.80–8.60] |
| **Liver damage** | 10.14 [6.46–15.91] | 3.20 [1.91–5.37] |

Abbreviations: CI, confidence interval; LDL, low-density lipoprotein; HDL, high-density lipoprotein.

Adjusted odds ratios and 95% CIs were calculated by logistic regression analysis using age, sex, and obesity score, future diabetes risk, hypertriglyceridemia, hyper-LDL-cholesterolemia, hypo-HDL-cholesterolemia, and liver damage as covariates.
Underweight was excluded from the table because this covariate had 0 events.
